# Understanding the Implementation of CareCoach—A Blended eHealth Intervention for Carers of People Living with Dementia: A Qualitative Process Evaluation Using Normalisation Process Theory

**DOI:** 10.3390/bs15081058

**Published:** 2025-08-05

**Authors:** Thando Katangwe-Chigamba, Margaret Guy, Jan R. Oyebode, Fiona M. Poland, Carl May, Chris Fox, Helen Morse, Jane L. Cross

**Affiliations:** 1 Faculty of Medicine and Health Sciences, University of East Anglia, Norwich NR4 7TJ, UK; t.katangwe@uea.ac.uk (T.K.-C.); margaret.guy@uea.ac.uk (M.G.); f.poland@uea.ac.uk (F.M.P.); helen.morse@uea.ac.uk (H.M.); 2 Centre for Applied Dementia Studies, Faculty of Health Studies, University of Bradford, Bradford BD7 1DP, UK; j.oyebode@bradford.ac.uk; 3 Faculty of Public Health and Policy, London School of Hygiene and Tropical Medicine, 15-17 Tavistock Place, London WC1H 9SH, UK; carl.may@lshtm.ac.uk

**Keywords:** dementia, carer, caregiver, process evaluation, implementation, coach

## Abstract

CareCoach seeks to enhance self-efficacy in family caregivers of people living with dementia and has been feasibility tested in a multicentre randomised controlled trial. The intervention offers two face-to-face sessions with a trained coach and access to an online platform with nine modules. This paper reports findings from an embedded qualitative process evaluation assessing implementation from the implementer’s (‘coach’s’) (n = 8) perspective using individual interviews and implementer group discussions. Qualitative data were transcribed verbatim, inductively coded and analysed using Normalisation Process Theory. Implementers demonstrated (1) ‘Coherence’ by seeking to understand how CareCoach compared to current practice, highlighting the importance of supporting coaches to differentiate and identify boundaries between their new ‘coach role’ and usual practice; (2) ‘Cognitive Participation’ by reviewing training and resources to understand their role own responsibilities and facilitate delivery of coaching sessions; group supervision and peer support were also emphasised; (3) ‘Collective Action’ through interactions with carers to deliver key behavioural aspects such as goal setting, problem solving, and providing feedback; and (4) ‘Reflexive Monitoring’ by appraising the intervention to gain useful insights that could facilitate refinement of CareCoach training and delivery. This study provides a theoretically informed understanding of the implementation of CareCoach for caregivers of people living with dementia and provides recommendations to enhance training for coaches, intervention delivery and carer engagement.

## 1. Introduction

Dementia is the seventh leading cause of death globally and is one of the primary causes of disability and dependency among older people (World Health Organisation, 2023). Dementia affects 982,000 people in the UK (Alzheimer’s Society, 2021a, 2021b), most of whom (700,000) are supported by family members or other untrained carers who do not receive any additional support (Alzheimer’s Association, 2014; Whitlatch & Orsulic-Jeras, 2018). This paper examines the implementation of a supported carer self-management intervention, seeking to enhance their self-efficacy and self-management to mitigate stressors raised for these otherwise unsupported carers. In this study context, implementation refers to processes and strategies adopted by coaches to facilitate the practical delivery of CareCoach (Skivington et al., 2021).

The experience of caring for people living with dementia (PLWD) has been closely associated with financial, physical, and emotional stresses arising from carers having to balance heavy demands of work, family and caring (Brodaty & Donkin, 2009; Schulz & Martire, 2004). Carer stress has been linked to their reduced productivity at work, mental health problems (e.g., depression, anxiety) and poorer physical health (e.g., arthritis, heart disease), leading to their increased use of healthcare services (Bosboom et al., 2013; Martín-García et al., 2016; Schulz & Martire, 2004; Wang et al., 2014).

Carers’ ability to adapt to the situation of supporting a person with a dementia diagnosis is often influenced by how they respond to such physical and emotional stressors (De Vugt & Verhey, 2013; Gallagher et al., 2011). Carers learning ways to focus on living positively despite a close family member having dementia, rather than focusing on the negatives of the dementia, may facilitate carers adjustment and adaptation (Boots et al., 2014). Interventions designed to help carers gain more positive caring experiences have been shown to improve carers’ wellbeing (Quinn & Toms, 2019) and to reduce or delay the need for carers to access health services (Egilstrod et al., 2019). Online interventions for caregivers of people with early-stage dementia have shown significant improvements in carers’ wellbeing, self-efficacy, mastery, and quality of life (Boots et al., 2018). When used together with support from health professionals, online interventions have been shown to increase access to support whilst bridging gaps in internet literacy and access to the internet (Boots et al., 2014; Dalton et al., 2018; Hopwood et al., 2018; McKechnie et al., 2014; National Institute for Health Clinical Excellence, 2018).

In the UK, the CareCoach intervention is an eight-week self-management programme for family [or unpaid] carers of PLWD. It offers two face-to-face sessions with a trained coach and access to an online platform with a selection of nine modules. This blended intervention has been co-designed (Scheibl et al., 2024) in response to the increasing incidence of dementia (Alzheimer’s Society, 2021b) and the lack of support for carers of PLWD (Whitlatch & Orsulic-Jeras, 2018). The intervention is an adaptation of ‘Partners in Balance’ (PiB), a theoretically based and evidence-based intervention designed in the Netherlands, shown to result in significant improvements in carers’ self-efficacy, mastery, and quality of life (Boots et al., 2018, 2016). CareCoach has been feasibility-tested in a multicentre randomised controlled trial to assess recruitment, retention and outcome data completion (Morse et al., 2024). This article reports findings from an embedded qualitative process evaluation, which aimed to assess implementation of the intervention from the implementers’ (coaches’) perspective. It uses Normalisation Process Theory (NPT) (May & Finch, 2009) to help conceptualise and specify the intervention in the action context. NPT provides a theory-building way to understand and evaluate implementation of complex interventions (May & Finch, 2009). NPT can be used flexibly with qualitative methods to conceptualise barriers and facilitators to embedding interventions in practice. NPT therefore guides an emergent understanding of the ways and extent to which the new practice has become normalised (i.e., as part of routine care). NPT has previously been used successfully in several studies involving people with dementia and carers to conceptualise the implementation of interventions (Kellett et al., 2023; Mathieson et al., 2020; McEvoy et al., 2014; Svedin et al., 2023). The NPT framework has four constructs: (1) Coherence—the sense-making work that people do individually and collectively when they are faced with the problem of operationalising some set of practices; (2) Cognitive Participation—the relational work that people do to build and sustain a community of practice around a new technology or complex intervention; (3) Collective Action—the operational work that people do to enact a set of practices, whether these represent a new technology or complex healthcare intervention; and (4) Reflexive Monitoring—the appraisal work that people do to assess and understand the ways that a new set of practices affect them and others around them. Each of the constructs has four corresponding components which guide examination and conceptualisation in the action context of how people have worked individually or collectively to enact a specific set of practices. This helps identify whether, where and how an intervention has or has not become a part of everyday practice and what factors may have facilitated or obstructed this.

The specific aim of this aspect of our wider study was to use NPT to understand how and how far the CareCoach intervention for carers of PLWD was implemented, evaluating the barriers and facilitators to its delivery. Drawing on the NPT framework, the objectives of the paper were (1) to explore coaches’ experiences of receiving training and delivering CareCoach and (2) to identify whether and how learning from the feasibility trial could be used to refine the CareCoach intervention, training and delivery. This process evaluation will address the gap in understanding how and why interventions for carers of people with dementia work (or don’t work), complementing future outcome evaluations that focus on whether an intervention achieved its goals (Moore et al., 2015; Skivington et al., 2021).

## 2. Materials and Methods

### 2.1. Study Design

A process evaluation of the CareCoach programme was used to assess the intervention implementation from the coaches’ perspective. The process evaluation employed a qualitative interpretive methodology focused on understanding meanings and interpretations that individuals give to their experiences and actions (Lim, 2024; Smith et al., 2025). This methodology was identified as suitable to explore both subjective and contextual nature coaches’ experiences in implementing CareCoach. Data collection methods included individual semi-structured interviews with coaches at the end of the intervention period and implementer group discussions conducted at different points during the intervention. Carers’ experiences of receiving the intervention have been reported elsewhere (Katangwe-Chigamba et al., 2024). The process evaluation was embedded within the CareCoach feasibility trial, a parallel, multicentre, individually randomised controlled trial (RCT) conducted in the UK between May 2022 and April 2024. The study received ethical approval from the National Research Ethics Committee (REC ref: 22/NW/0293) and was registered on a clinical trial registry (ISRCTN12540555).

### 2.2. The CareCoach Intervention

**Figure 1:**
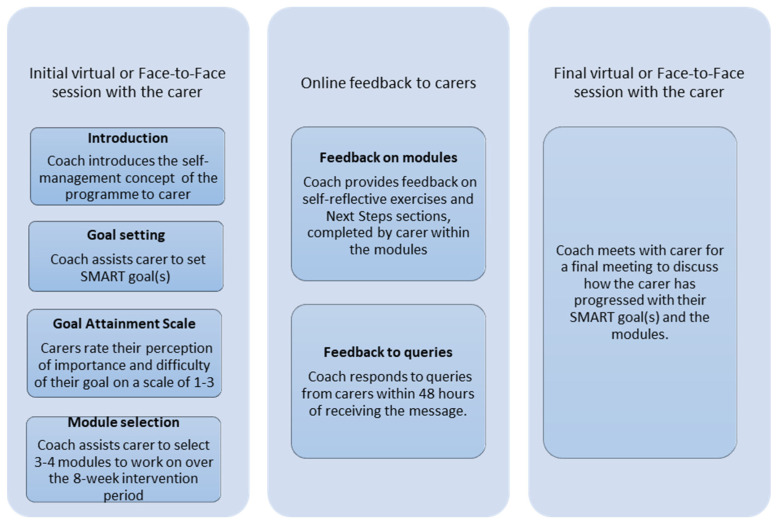
CareCoach intervention delivery process.

The CareCoach programme is described in detail elsewhere (Fox et al., 2021). Briefly, CareCoach is an 8-week self-management programme comprising three core components: (1) two virtual sessions with a personal coach at the start and end of the intervention to introduce the intervention to carers, assist them to choose relevant modules and set SMART goal(s) to complete the Goal Attainment Scale (GAS) (Jennings et al., 2018) (each goal rated by the carer on a scale of 1–3), review progress and discuss next steps; (2) an online platform giving carers access to nine learning modules, from which carers choose the four most relevant to their situation; (3) online feedback from a coach using the online message function on the online platform throughout the intervention period. A summary of intervention delivery process and work done by coaches during the intervention period is detailed in Figure 1.

All coaches attended up to two hours of training to use the intervention, how to provide feedback, how to support caregivers to set SMART goals and select modules, and how to use the Goal Attainment Scale. The training was supported with several resources, including the CareCoach manual, a 28-page guidance document providing information on the background and objectives of CareCoach; the role of the coach; the coaching sessions; SMART goals; the Goal Attainment Scale (GAS); giving feedback to the carers; detailing the modules; and other general tips. This training was also appraised by the coaches as part of our evaluation.

Coaches attended fortnightly group supervision sessions with a clinical psychologist where they had the opportunity to express concerns, ask questions and share their experiences with other coaches.

### 2.3. Study Setting and Participants

Participants came from three sites at NHS Foundation Trusts (providers of dementia care) and one at a research institute for the care of older people which maintained a register of carers keen to take part in research studies. They were in diverse areas, covering rural and urban, white British and multi-ethnic populations in the North and South of England. Participating coaches delivered the intervention to carers of PLWD from their NHS Foundation Trust and/or to carers recruited remotely from other NHS Foundation Trusts. Eligible carers were over 18 years of age and caring for a PLWD diagnosed within the last five years with whom they had an immediate family relationship (spouse/partner, sibling, son or daughter) or close personal relationship (e.g., other family member or close friend).

### 2.4. Data Collection

Semi-structured online qualitative interviews with all eight active coaches were used to explore their approach to coaching, their experiences of coaching sessions and using the web-based platform, and their thoughts on the training provided for CareCoach. The interviews were facilitated by a topic guide (see Supplementary Materials) and explored coaches’ perceptions of carers’ views of the intervention. The interviews were conducted by two researchers with no prior involvement in intervention development (TKC and MG) using a topic guide structured around the four NPT constructs. In addition, three online implementer meetings were held during the intervention delivery period to enable coaches to discuss their experiences in a group setting. Implementer meetings were unstructured meetings (facilitated by JC and TKC) designed as a space for coaches to share their experiences and problem solve together. The meetings took place at six-week intervals during the six-month intervention delivery period of the feasibility study.

### 2.5. Data Analysis

All interviews and implementer meetings were recorded with consent, transcribed verbatim, anonymised, and uploaded onto NVivo^®^ 15 software for analysis. All transcripts were initially inductively coded by TKC and MG before synthesising the data by mapping relevant codes to the core NPT constructs: Coherence; Cognitive Participation; Collective Action; and Reflexive Monitoring. Inductively coding the transcripts ensured that the researchers had a thorough and unbiased understanding of the data before applying it to the NPT framework (Skjott Linneberg & Korsgaard, 2019).

Following initial coding and the first round of mapping, multiple analysis meetings were held with the study team, to refine and further develop the findings, relating them to NPT constructs. This provided a way to conceptualise and evaluate coaches’ experience of both training for and delivering the intervention to help judge whether CareCoach needed refining.

## 3. Results

Eight coaches took part in interviews, seven of whom attended at least one of the three implementer meeting(s). Coaches were from a range of occupational roles, including assistant psychologists and research assistants (see Table 1). All provided the intervention for local carers while two also provided coaching for remote carers.

The findings are presented within the four constructs and related sub-constructs of the of the NPT framework.

### 3.1. Coherence

In examining coherence here, we focused on understanding the sense-making work done by coaches, individually and collectively, to implement CareCoach as a set of practices. This sense-making work is reported in terms of themes representing the sub-constructs of Coherence (see Table 2).

#### 3.1.1. Understanding the ‘Coach Role’: Differences and Similarities with Usual Practice and Concerns About Overstepping Boundaries (NPT Sub-Construct—Differentiation)

The sense-making work done by coaches to understand how CareCoach is different from their usual role involved understanding the role of the coach. The role of the coach required understanding carers’ situations, guiding them through problem solving and goal setting and encouraging behaviour change; this was ‘new’—and therefore different—to most coaches. Coaches articulated such differentiation by drawing on similarities and differences between CareCoach practices and their usual, sometimes therapeutic practice. Similarities included journaling or reflecting on emotions or setting goals:

Most of the tips felt very relatable to what we were doing like in therapeutic work with someone. So, for example, journalling your thoughts or giving yourself time to experience your emotion

[Coach01]

Even where similarities were identified, coaches could differentiate further by identifying boundaries between the coach role and their usual role. For example, coaches with a therapy background highlighted the challenges of not taking on a ‘therapist role’ during the sessions by not giving carers advice or exploring carers’ feelings in depth. For some, identifying boundaries between the ‘coach’ and ‘usual’ role also raised challenges for possibly overstepping boundaries.

It has been a bit difficult to set aside, to establish the boundary between working therapeutically with someone and being a coach. I don’t know if I overstepped some boundaries, but it felt better to validate first.”

[Coach01]

Most coaches, understanding their role in CareCoach to be that of guide rather than giving direct advice, reported having to adjust from their usual role. Some coaches therefore went on to attempt to redefine their new role to make sense of the set of guiding practices they needed to operationalise engaging and supporting CareCoach:

It was obviously a new angle […] you need to guide people, not tell people what to do. In my nurse role sometimes you’d be like “You can try this […]” So that’s an adjustment because I’ve got to just step back and let them take the lead.

[Coach07]

#### 3.1.2. Understanding CareCoach as a Self-Management Programme (NPT Sub-Construct—Individual and Communal Specification)

Beyond attending training, the sense-making work done by coaches individually to understand their specific tasks and responsibilities (as individual specification) primarily involved referring to notes they took during the training and reviewing the manual. In fact, some coaches reported the training as only making sense after they had gone through the manual.

Specifying CareCoach also involved coaches working together with carers to build a shared understanding of the aims, objectives and expected benefits of CareCoach (*Communal specification*). Communal specification involved aligning coaches’ and carers’ understanding of the self-management concept of the CareCoach programme. The training helped most coaches understand CareCoach to be a self-management programme, which they then communicated to carers during the sessions.

Where carers clearly understood and accepted CareCoach to be a self-management programme, this facilitated them to implement activities such as problem solving and goal setting. Coach02 related this to providing “background” support and an “informative programme” for carers to achieve their goals.

She really understands that she has to do it by herself, but I’m there very in the background just to support her, but I’m not going to be able to give her answers.

[Coach02]

However, coaches reported that some carers’ understanding and expectations conflicted with the self-management concept of CareCoach in continuing to expect advice and solutions from the coach, rather than as empowered learners to solve their own challenges. This expectation impeded implementing practices such as goal setting and problem solving, which had to be carer-led. The coaches therefore highlighted the importance of making explicit to carers what CareCoach involves earlier on, less about “being a better carer”, and more about self-managing “their own wellbeing” [Coach04]. Coach06 emphasises more active direction finding through, e.g., signposting, rather than seeking advice from the coach.

[Carer name] thought it was going to be having somebody to talk to, to give them advice. I really emphasised that my role isn’t so much to give them advice but more to I guess signpost them towards any support and also get them to reflect and think about what they can do

[Coach06]

#### 3.1.3. Understanding the Value of CareCoach (Internalisation)

Finally, CareCoach *internalisation* involved coaches reflecting on the specific value and benefits of CareCoach for carers of PLWD. Coaches saw CareCoach as a good first step for supporting carers in the early years after diagnosis and when they could look at things differently so as to manage their role better. The involvement of support from a coach to help carers problem solve and set goals through helping them “shift their focus to themselves” was viewed as a novel aspect of the programme.

It’s helping people to maybe look at things from a different perspective. […] the idea that, […], the programme can give them a bit more information and you can maybe guide them into making the most of that and also taking that time to really focus on themselves and giving them a bit more headspace to process some of the stuff about the way they’re coping

[Coach03]

Coaches highlighted benefits of the blended approach to delivery as providing carers with flexibility to access support when it was convenient for them. However, as the programme was to be self-managed, coaches felt carers who are self-motivated would benefit most from CareCoach.

I think because it’s a self-management programme, as a coach you can only do so much. Someone has to be self-motivated, and they have to want to help themselves.

[Coach04]

### 3.2. Cognitive Participation

Cognitive Participation focused on understanding the relational work that coaches did to build and sustain a community of practice around the CareCoach complex intervention. This relational work is reported as themes representing the four sub-constructs (see Table 3).

#### 3.2.1. Beyond Training: Coaches Organising and Reorganising Themselves and Others to Deliver CareCoach Sessions (Enrolment)

The work done by coaches to organise themselves to deliver CareCoach following training initially involved reviewing training documents, familiarising themselves with the contents of the online platform, and going through the manual. Coaches’ individual organisation also involved creating summaries/checklists that would help facilitate delivery of the initial session with carers. Coaches reported using various resources, including the coaching manual, coaching guidance and reflection sheet, as checklists to structure the sessions. This gave them specific terms to use to identify key activities and information to share in sessions

After the training, when I got access to the platform, again, I spent a lot of time going through the modules, writing up summaries so I can get an idea about the modules, and then preparing my own bullet points for my first interview with the participant

[Coach02]

For some coaches, getting organised involved working with others to create templates to help structure session delivery or sharing tips on ways to structure the sessions. For example, two coaches working in the same organisation created a template with these steps: finding out what the carers know about CareCoach; introducing CareCoach; carers background and experiences; selecting modules; goal setting; and scoring goal attainment. Another coach reported adopting a fellow coach’s checklist after attending a supervision session.

Me and [Other coach] have created a CareCoach script of how we want the appointment to go ideally, or how to structure it. So that helped a lot, because even though I’ve done seven initial interviews, I’ve used it throughout, just to make sure that I’ve done it

[Coach01]

Although having a structure for delivering the sessions was seen as important, most coaches remained flexible to ensure sessions remained based on carers’ priorities and needs.

#### 3.2.2. Time Requirements to Build and Sustain CareCoach Implementation (Initiation)

Coaches reported spending varying amounts of time to prepare for the initial session (30 min/afternoon). To deliver the initial session, coaches worked flexibly (initial session: 40–120 min) to match carers’ IT skills, availability and prior knowledge.

I was spending anywhere between an hour-and-a-half to two hours, with people for the initial interview. I do think it’s important to spend that amount of time […] for rapport-building

[Coach04]

She was really proactive, and she was very tech-savvy that’s why […] our session didn’t take the full 60 min

[Coach05]

Sustaining CareCoach implementation beyond the initial session involved coaches responding to carers’ feedback to guide them through selected modules. Coaches reported spending anywhere between 10 min and 2 h to provide online feedback to carers, depending on their confidence and experience in responding to feedback. However, some coaches highlighted as a particular challenge to sustaining the implementation, the requirement to respond to carers’ feedback within 48 h.

So the first time […] I was being quite careful about my phrasing. I took […] maybe 30 min because I was definitely second-guessing myself […]. It’s got quicker with providing feedback. So the most recent one probably took me maybe 10 min to write.

[Coach03]

#### 3.2.3. Am I Right for the Role? Coaches’ Legitimation of Their Involvement in Delivering CareCoach (Legitimation)

Legitimation, an important contributor to participating, here hinged on whether coaches believed that it was right for them to be involved in delivering CareCoach and their contribution would fit. Some coaches, reflecting on their professional background and experience (e.g., working with people with dementia) felt the coach role fitted well with their usual role and interests (e.g., working with older adults). These coaches saw coaching as an opportunity to make a valid and worthwhile contribution in supporting carers and felt motivated especially when they saw carers progress, achieve goals and feel empowered to manage their situation.

That feeling that I’ve got after I’ve had a final interview with someone and it’s been quite a positive one, it’s really given me a bit of a buzz for the rest of the day. It’s made me feel like what I’m doing is worthwhile, and that’s really rewarding

[Coach04]

Other coaches, however, reflecting on differences between the coach role and their professional background and experiences, expressed less confidence to undertake the role. These coaches reported perceived limitations, including a lack of experience or clinical experience of working with people with dementia.

I don’t feel really confident, to be honest, because I don’t have the clinical experience, I definitely feel that it’s something that I always have to go back to my line manager

[Coach02]

As such, these coaches’ narratives regarding their role in delivering CareCoach were marked with concerns about delivering aspects of the intervention correctly. For example, coaches worried about how to offer a suitable type of feedback and that they used a suitable tone in such feedback to carers during the intervention:

I’m really struggling to make sure that whatever I say, I don’t sound patronising, or I don’t sound too friendly or too formal or very repetitive like a robot

[Coach02]

I’m really careful not to sound like a schoolteacher marking their homework and maybe sound a bit patronising. Or […] phrase that in a way that doesn’t sound like I’m telling them off

[Coach03]

#### 3.2.4. The Role of Supervision Sessions and Other Support in Sustaining the Delivery of CareCoach (Activation)

Supervision sessions were highlighted as an important support for enabling coaches to sustain the delivery of CareCoach beyond initial training. Benefits of supervision included providing a space for sharing challenges and acquiring coaching techniques and solutions to challenges.

The group element was highlighted as important in allowing coaches to learn from each other’s experiences and share practices such as creating scripts for delivering the initial session. One coach mentioned how they implemented an approach to goal setting after receiving advice from another coach.

Someone mentioned, if you find it difficult to come up with a suggestion, you can just ask, “What would you like to gain out of the intervention in general?” I think that was very smart to use as a question, as a prompt

[Coach01]

Peer learning through supervision was highlighted as beneficial even before some coaches started delivering the sessions, as this gave them a chance to prepare for the session and adjust their expectations. The support also helped increase coaches’ confidence and reduce worries about how to provide relevant feedback, freeing them to effectively implement CareCoach.

That was actually hugely helpful because we were starting to hear the questions that were coming up or feedback from others who were already doing it. So that was very useful to hear before we started, because we picked up a few things from that, as well.

[Coach03]

Other forms of support, helping sustain delivering CareCoach especially where coaches lacked confidence in providing feedback, came from principal investigators, line managers, research team or supervisors in their workplace.

With the guidance through the supervision and […] support from the study team and my line manager, the PI, I think I really understood how to put together my answers and my feedback […] without worrying that much.

[Coach03_implementer meeting]

Most workplace supervisors had a psychology background or experience with working with people with dementia which coaches found helpful in addressing concerns they had (e.g., determining boundaries). However, coaches who had supervisors without psychology background expressed limitation in support beyond the group supervision.

My line manager, she’s also the PI, because her background working with people with dementia really helps for me to double-check with her if whatever I said to the carer makes sense.

[Coach02]

Where I am we don’t have a clinical psychologist so beyond the group supervision I don’t have that level of supervision.

[Coach06_implementers meeting]

### 3.3. Collective Action

Collective Action focused here on conceptualising the operational work that coaches did to enact the implementation of the CareCoach complex intervention. This operational work is reported as themes representing the three sub-constructs of Collective Action (see Table 4).

#### 3.3.1. Operationalising CareCoach: Delivering Sessions and Maintaining Carer Engagement (Interactional Workability)

The work done to operationalise CareCoach involved coaches using resources such as the manual to explain the aims and objectives of the intervention to carers. In addition, some coaches found the suggested prompts in the manual useful for engaging carers to approach activities purposefully when interacting with them.

I really liked how, on the manual, there were specific prompts for the coaches to use when replying to carers. Having that there helped me to reflect on my responses and how I would approach a discussion and how I would work towards a goal

[Coach01]

Here, Coach01 felt resources better informed them to share and discuss goals in engagement. In the initial session, operationalising the intervention also involved coaches and carers working together to set SMART goals and select modules. To operationalise these relational tasks, coaches gave pre-eminence to building rapport with carers and developing a good understanding of their situation before selecting modules and setting goals.

I asked some really basic questions at the start to get to know each other better. To share about her experience of the person with dementia, when it started…Also, how that’s impacting her and what’s changed for her really since the person was diagnosed.

[Coach05]

Goal setting and module selection were primarily carer led, whereas coaches’ involvement was ensuring that selected goals were SMART or highlighting modules that could be useful. This aspect was highlighted as most challenging to coaches, i.e., to ensure that the goal was achievable and measurable. Less commonly, module selection and goal setting were led by the coach, based on knowledge of each carer’s background. This usually occurred when carers were struggling to take the lead themselves and time constraints were placing the dyad under pressure. For some coaches, the responsibility of selecting modules on behalf of carers was worrisome.

I went away after the session…I took it back to my own supervisor and was just trying to make sense of what we had discussed. Then what I did from that was send it back to her and made it into a SMART goal

[Coach05]

So when I’m going to be ready to show them the modules I will be like, “I would suggest this and this and this module are appropriate for you, what do you think?” for me it’s still really scary because my feedback, by accident, can have a negative impact by maybe allocate them to the wrong module

[Coach02]

Coaches highlighted several challenges with the relational work of goal setting including lack of engagement from carers. This included having primary motivation for enrolling in the study simply being to contribute toward research; finding the contents too easy; struggling with the concept of SMART goals; or setting unrealistic goals. Coaches reported using different methods to help carers realise that goals set were unrealistic.

He said, “I want to be able to tell my wife not to do something without her being upset.” I talked a bit about what it is about his communication, and he agreed that he did have some issues in communication, and he used a very authoritative voice. So he wanted to improve his communication by thinking about his language and his tone of voice when responding to something that his wife has done that isn’t to her usual standard.”

[Coach06]

In this quote, the coach ‘unfolded’ the husband’s goal, helped him think about what influenced his wife’s upset and ways he could change his behaviour so that she is less likely to become upset. Initially, coach/carer dyads attempted to set one goal per module. However, the challenges of ensuring that goals were SMART led to revising this alongside the research team to set one overarching goal for participating in CareCoach, which coaches reported as more manageable.

Following the initial session, maintaining engagement with the modules was a key challenge. A few coaches reported some carers lacked engagement with five-step planning, did not respond to feedback or rushed through the modules:

They haven’t been very engaged with the step-planning process, but they are completing the modules. When she’s filling in the action plans and all those sections, she will sometimes just write ‘not relevant to me’ in the boxes. So, there’s a lack of engagement there because she doesn’t want to focus on herself, which is what all of it is asking you to do.”

[Coach03]

Where there was a lack of engagement, some coaches adopted a mindful approach by using occasional prompting to ensure that were not overburdening carers. Other coaches adopted an active monitoring approach by maintaining regular contact to prompt and encourage carers to complete the modules and even offered additional meetings if needed.

When there was no activity for the two weeks, and when I emailed just to be like, “Is everything OK?” I think that did really prompt her to do it…maybe if she hadn’t got a check-in, it could have slipped her mind”

[Coach05]

#### 3.3.2. The Role of Peer Support and Experience in Maintaining Confidence in Implementing CareCoach (Relational Integration)

Relational Integration, the knowledge work coaches did to build accountability and maintain confidence in implementing CareCoach, mainly involved peer support and experience. Here, such support involved coaches shadowing each other and asking advice from each other.

The fact that it wasn’t just me in my site as a coach, we shadowed each other just to see what we were doing, and that was really useful.

[Coach04]

Some also reported colleagues bringing experience from other areas such as dementia studies to inform some similar activities. Coaches reported how gaining experience increased their confidence in delivering CareCoach sessions and providing feedback.

I think to begin with I had quite a similar experience in terms of kind of finding my feet and trying to figure out how to respond to people and stuff. But I think yeah once you’ve had a bit of practice and got in the rhythm of it I felt a lot more confident with it.

[Coach04_implementer meeting]

### 3.4. Reflexive Monitoring

Reflexive Monitoring focused on identifying and conceptualising coaches’ appraisal of the work they did to assess and understand the ways that implementing the CareCoach intervention affected them and the carers. This appraisal work is reported as themes representing the four sub-constructs of Reflexive Monitoring (see Table 5).

#### 3.4.1. CareCoach—Empowering Carers to Manage Their Role and Improve Their Wellbeing (Systematisation)

Most coaches, drawing on carers’ experiences and feedback, found CareCoach to be a useful intervention for empowering carers’ managing skills. Highlighting skills such as goal setting, coaches reported the intervention as empowering carers to manage their situation and focus on their wellbeing. Other skills carers learned, such as communication and problem-solving, were seen as having had a wider impact on carers relationships beyond those with the PLWD. This is exemplified by Coach02 identifying a participant as now able to accept a situation while minimising distress it might cause

She found the goal setting system really helpful for her life in general. [And] how sometimes to accept that maybe something is not going to change but how to minimise the distress that this situation is causing to her

[Coach02_implementers meeting]

#### 3.4.2. Coaches’ and Carers’ Evaluation of CareCoach (Communal Appraisal)

Communal appraisal of CareCoach by carers and coaches was included in the final session and highlighted several factors as playing a role in how carers perceived the impact of the intervention. Firstly, coaches saw carers’ openness to learn and develop new skills as depending on whether they could relate the content of modules and videos to their situation. For example, some coaches reported that some non-spousal carers found videos and module content did not resonate with their situation and so less helpful.

They did comment on the videos being very focused on spouse examples and some of the content assuming that you might be living with a person […]. For them it was quite different, they’d visit once or twice a week and speak on the phone. So, there were aspects that meant they found it less helpful

[Coach08]

Secondly, the impact of CareCoach on carers’ knowledge was reported to have depended on their background knowledge of dementia and psychoeducation concepts with the modules.

I think she’s quite switched on in terms of psychology and this stuff. So I think some of the modules […] seemed quite basic and quite simple

[Coach04]

Thirdly, carers who perceived the content of CareCoach to have validated their experiences were more open to learning and found the intervention useful, whereas carers who perceived their behaviours and reactions to have been criticised or judged by the content of the intervention or who did not agree with suggested ways of responding to the behaviours of the PLWD (e.g., not arguing back) found the intervention unhelpful.

This was the person that found it quite condescending. I think she took umbrage to the fact that she felt that the programme was saying that the carer is doing something wrong, and they need to change what they’re doing. But I think she also just generally had some issues with the way that dementia is treated in society.

[Coach04]

#### 3.4.3. CareCoach Training and Delivery Enriching Coaches’ Usual Practice (Individual Appraisal)

Most coaches reported benefiting from being involved in CareCoach and highlighted acquiring several skills, including building rapport with carers and learning to empower people without offering solutions or using psychotherapeutic techniques.

I’ve learnt how you can coach someone and still support them without using, for example, a CBT module or psychotherapeutic modules to help empower a person.

[Coach01]

The content of the modules could also be seen to equip coaches with knowledge applicable in their usual practice with other carers and people living with dementia. Some coaches viewed the blended approach to intervention delivery as a new experience and as useful for future interventions.

Just flicking through the modules, there were some things there that I may have not picked up yet from my role and might be helpful for me to apply in my appointments with other patients and their carers

[Coach05]

#### 3.4.4. Coaches’ Appraisal of the Blended Approach to CareCoach Delivery (Reconfiguration, Sub-Theme 1)

The blended delivery of CareCoach, involving carers accessing an online platform and receiving coaching sessions delivered via Zoom or Microsoft Teams, raised several IT barriers, including technological challenges with logging on/setting passwords and carers being unable to use online meetings packages.

Coaches reported using varied strategies to support session delivery to carers who struggled to use online platforms, including creating templates on how to use Teams or delivering the sessions over the phone.

I created a step-by-step of how to get onto the Teams call. After I did that I don’t think I really had anyone that had such an issue with it.

[Coach04]

However, coaches who delivered the intervention by phone identified challenges for building rapport, suggesting the need to incorporate in-person contact for carers who struggle with technology.

I think that [delivering the session over the phone] did make it a little bit more difficult… without that facial feedback that you get on a video call.

[Coach03]

Whilst most coaches found the eight-week programme duration to be acceptable, there were mixed views regarding the benefits of offering a mid-way meeting to carers between sessions to check on carers’ progress. In the following quote, Coach04 comments on how such contact might convey mixed messages about self-support.

I didn’t really feel the need to check in with people or have a mid-way [session] I suppose the way I see it as the least contact you can have the better—you don’t want them to be leaning on you too much, you want them to be able to support themselves.

[Coach04]

### 3.5. Coaches’ Appraisal of Training, Resources and Support Structures (Reconfiguration, Sub-Theme 2)

Coaches appraised the training and resources available for them to deliver CareCoach as mixed. Those with previous coaching experience found the training of up to two hours sufficient for the role and the manual comprehensive. These coaches highlighted ways they found the description of the coach role, the Q&A section in the manual, the reflection sheets and the CareCoach examples as useful.

I do remember it being really good. I actually referred back to the trainings and all the documents that were provided. So I do think they covered everything I needed to know

[Coach05]

Other coaches saw the training as too brief and the manual structurally poor, making it difficult to find information for delivering sessions. As such, to implement the intervention, these coaches reported using contents of the manual to create their own slides or templates to help structure the coaching sessions.

I don’t have the clinical experience. The training that we receive didn’t really feel enough. It was just one hour […] The manual, I think it’s all over the place. They have a few things about the first session […] the first few pages. There are a few things at the very end of the PDF. So, yeah, it took me time to put them together in order.

[Coach02]

These coaches identified a need for more specific support and guidance for delivering the initial and final coaching sessions to help to stay focused.

I think there were a few gaps just in terms of maybe just what the actual sessions should cover when you’re speaking to participants. That was left very open, and I think there could be a bit more guidance for the coaches in terms of what to cover

[Coach03]

Suggestions for refining guidance for delivering sessions included providing video recordings or transcripts of coaching as templates and deploying peer support by allowing coaches to shadow and learn from each other. Coaches also suggested it would be helpful to have the manual before the training, since the training makes more sense after going through the manual.

It might have been useful [to have] either a video or a transcript from a conversation of a coach and a participant just to give an example of all of those different points that you need to include

[Coach06]

Some coaches highlighted the need for more guidance for carers for setting SMART goals, several suggesting more guidance was needed on who should conduct the Goal Attainment Scale and when.

The sections in each of the modules where you set goals online, I wonder whether that might be maybe not clear enough, exactly what you’re supposed to do. Because you talk to the participant about SMART goals during the first appointment, but then it’s not necessarily very clear that that’s what they’re supposed to be doing in the modules because the questions, don’t reference any of the SMART stuff.

[Coach04]

Coaches’ involvement beyond the initial session involved them providing online feedback to carers. When appraising what is needed to help coaches to provide feedback, coaches reported the need for more training on coaching techniques (e.g., motivational interviewing) and more templates and examples of patient-centred prompts that could be used to validate carers experiences. Findings also highlighted the potential usefulness of templates of coaches’ responses to help respond to carers in different situations.

For discussions you might have [it would have been good to have] replies you might give in different situations -common themes that come up and how to respond to those, I think, maybe would have been helpful.

[Coach03]

Coaches’ appraisal of supervision sessions, a support structure most found helpful, was reported to be time-consuming, but a coach’s suggestion relevant here was therefore to offer fewer supervision sessions so that coaches only attend two supervision sessions a month, one facilitated with a clinical psychologist and another self-facilitated by coaches.

I don’t think I anticipated them [supervision sessions] being weekly. I mean they were really helpful so that was good. But, yes, it did take longer than I expected.

[Coach07]

In addition, some coaches, reflecting on the 48 h commitment to provide feedback to carers, suggested the need for more support structures for carers who may have varying availability. Coach04 suggested this could include offering diverse types of professional support.

I mean I think that would be helpful maybe once a month with a psychologist—yeah so I guess in a month you’d have one with a clinical psychologist and one just as a group of coaches?

[Coach04_implementers meeting]

## 4. Discussion

**Figure 2:**
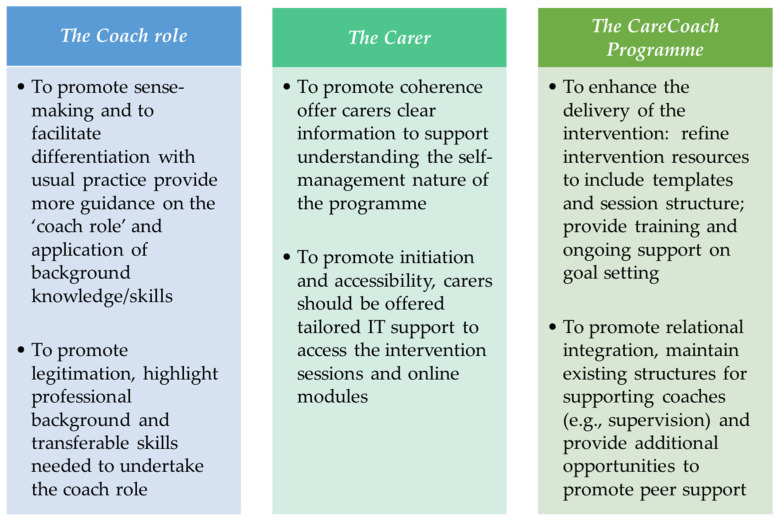
Key recommendations from study findings.

This study sought to understand the implementation of the CareCoach programme for caregivers of PLWD by using an NPT approach to explore coaches’ work to understand the intervention compared to current practice (Coherence); how they engaged (Cognitive Participation), operationalised it (Collective Action) and appraised it (Reflexive Monitoring) (May & Finch, 2009). A summary of key recommendations from our findings can be found in Figure 2.

Our findings provide insight into key sense-making work that coaches do when tasked with implementing CareCoach, particularly in differentiating the coach role from their usual practice. Coaches need support to understand the differentiation which could promote intervention fidelity, i.e., the extent to which an intervention is implemented exactly as intended by its developer (Moore et al., 2015; Skivington et al., 2021). In addition, gaining a clear understanding of the boundaries associated with the coaches’ “new” role appears critical for empowering carers to self-manage, particularly where coaches had diverse professional backgrounds and experiences of working with PLWD or carers and clinical therapeutic experience. This appeared to play a positive role in legitimising and further drawing on coaches’ background and experience for implementing the CareCoach intervention, which raises further questions regarding what sort of professionals, at what grade, may be best positioned to provide the intervention. This could inform cost-effectiveness judgements as employing people without specialist knowledge or experience may not actually deliver the specific intervention as intended.

Understanding the self-management concept of the programme was also identified as key sense-making work which involves both individual specification (by coaches) and communal specification (involving coaches and carers). This resonates with our parallel study which highlighted how some carers misunderstood the self-management concepts of the programme and the role of the coach (Katangwe-Chigamba et al., 2024). Misaligning carers’ expectations with the ethos of the programme may influence their engagement with the programme and the outcomes (Katangwe-Chigamba et al., 2024). Early communication with carers should therefore sufficiently designate and distinguish both the role of carer and of the coach in implementing and engaging with CareCoach (Rees et al., 2021).

Our findings therefore specify and contextualise the relational work needed to build and to sustain the implementation of CareCoach. Activities undertaken by coaches to prepare for the delivery of the intervention, including creating templates and checklists to structure the delivery of coaching sessions, provides useful learning that could be used to refine resources to support the delivery of CareCoach sessions. Group supervision, consisting of both professional (psychologist) and peer (other coaches) support was also identified as a vital enabler for sustaining the implementation of CareCoach. Supervision sessions providing coaches with a space to share challenges and receive advice and feedback were identified as helping to build confidence. This is in line with other psychosocial interventions which also identified supervision as important for developing an understanding of interventions and building confidence for those involved in delivering them (Finch et al., 2020; Maciag et al., 2023).

This study identifies the work of operationalising CareCoach as consisting primarily of interactions between coaches and carers to execute self-management activities such as problem solving, goal setting and module feedback. Our findings, suggesting goal setting to be a challenging task for both coaches and carers, underscores previous findings on carers’ engagement with CareCoach (Katangwe-Chigamba et al., 2024) and PiB (Boots et al., 2016). In line with previous research, we highlight key barriers to goal setting as carers attempting to change behaviours of PLWD rather than focusing on enhancing positive caring experiences (Boots et al., 2016); setting multiple goals as part of the initial session and modules (Baker et al., 2021; Katangwe-Chigamba et al., 2024; Mercer et al., 2016); and caregivers having unrealistic expectations of what are achievable goals (Bennett et al., 2011; Levack et al., 2006). Building on this, this study further identifies techniques that coaches could use to set SMART goals, e.g., starting off with a wider goal before breaking it down into smaller, achievable goals (Levack et al., 2006) or using questioning techniques to help carers come to the conclusion that goals set to change behaviours of PLWD may be less realistic than gaining skills to actively improve experiences of caring.

Our findings emphasise the importance of coaches’ skills and background in operationalising CareCoach. We found that some coaches draw on other therapeutic skills, including motivational interviewing and validation when delivering the intervention. Therefore, to ensure fidelity of the intervention whilst maintaining diversity of coaches, it will be important to take time to clarify flexibility for coaches to deploy previous skills when delivering the sessions and to provide additional training where needed for those without previous therapeutic training and experience.

Finally, the reflective joint appraisal work done by coaches to evaluate CareCoach provides insight into the benefits of the intervention and how to refine it. These highlighted that CareCoach was seen as empowering carers to manage their role. This fits with the proposed programme theory of the intervention (Boots et al., 2018; Scheibl et al., 2024). This research also highlighted wider benefits for coaches from CareCoach training and delivery, suggesting that identifying skills to be learned by coaches could be useful for delivering other carer interventions. Further research could therefore explore the wider impact of CareCoach training and delivery for developing and delivering other interventions.

Importantly, our findings highlight key recommendations that could enhance the delivery of CareCoach, including providing additional resources to help coaches structure intervention sessions and to offer online feedback to caregivers; additional training on coaching techniques, e.g., motivational interviewing; and more specific guidance with delivering the first and final coaching session (Katangwe-Chigamba et al., 2024).

Our study showed that while the blended approach to delivery used here has been identified as an accessible way of delivering support for carers (Boots et al., 2016) but still presented barriers for some carers who were not technologically savvy (Katangwe-Chigamba et al., 2024). However, our findings have helped specify ways to support carers who are not technologically savvy to ensure both equity and accessibility of the programme. Adaptations used or suggested by coaches in this research included using templates on how to access platforms such as MS Teams or providing in-person meetings. These could be used to adapt future delivery of CareCoach to enhance the experiences for those carers who struggle with using technology.

### Strengths and Limitations

This qualitative process evaluation focuses on conceptualising and identifying the implementation of the CareCoach programme. Using NPT has provided a robust and well-connected framework for evaluating complex interventions for caregivers (Moore et al., 2015; Skivington et al., 2021). The NPT framework, which provides a comprehensive explanation of the constituents of implementation processes (Nilsen, 2015), was considered appropriate to facilitate detailed learning required for refining CareCoach at the feasibility stage of the study. Other implementation frameworks (e.g., RE-AIM and CFIR), which consider other aspects of implementation including reach and effectiveness, are more appropriate for the definitive stage of evaluating complex interventions (Moullin et al., 2020; Nilsen, 2015).

Including multiple sources of qualitative data (interviews and implementer group discussion) provided comprehensive, specified, staged and contextualised understandings of actions and resources used in implementing CareCoach (N. Carter et al., 2014). In addition, including both interviews and group discussions provided an in-depth understanding of the implementation process whilst allowing for opportunities for reflection based on coaches’ experiences. While our findings provide initial insight into how CareCoach is implemented, the small sample size may limit wider representativeness of our conclusions, but the detailed descriptions and accounts this approach provided can enable transferability. A key challenge of using the NPT was the overlapping nature of the construct, meaning that data were mapped to more than one construct (H. Carter et al., 2023). Additionally, the use of NPT provided a structured and theoretically informed approach to conducting the process evaluation (Moore et al., 2015; Skivington et al., 2021), whereas a non-framework approach may have led to more diverse and novel insights and understanding (Bonner et al., 2021).

## 5. Conclusions

This study provides a theoretically informed understanding of the engagement of coaches and caregivers of PLWD in implementing the CareCoach intervention. Our findings highlight key features that could enhance how coaches deliver the programme. These include offering more support to help them differentiate the coach role from usual practice and support to build carers’ understanding of the self-management concept focusing the intervention. Our findings also highlight the importance of group supervision and peer support in sustaining implementation of the intervention. Our findings highlight key recommendations that could enhance delivery of CareCoach. These include providing resources to help structure delivery of the intervention sessions, training on coaching techniques and templates to help with providing feedback.

## Figures and Tables

**Table 1:** Professional background of participating coaches.

Characteristics		Coaches (n = 8)
Coaches’ job role	Research assistant psychologist	3
	Assistant psychologist	1
	Research psychologist	1
	Research nurse	1
	Senior research assistant	2
Site delivery method	Locally	6
	Remotely	0
	Both locally and remotely	2

**Table 2:** Definitions and themes found for sub-constructs of Coherence.

NPT Sub-Constructs	Definition	Themes
Differentiation	The sense-making work done by coaches to understand how CareCoach, as an intervention and a set of practices, is different from their usual practice role	Understanding the ‘coach role’—differences and similarities with usual practice and concerns about overstepping boundaries
Individual and communal specification	The sense-making work done by coaches and carers to understand their specific tasks and responsibilities here	Understanding CareCoach as a self-management programme
Internalisation	The sense-making work done by coaches to understanding the value, benefits and importance of CareCoach	Understanding the specific value, benefits and importance of CareCoach

**Table 3:** Definitions and themes found for sub-constructs of Cognitive Participation.

NPT Sub-Constructs	Definition	Themes
Enrolment	The work done by coaches to organise and reorganise themselves and others to deliver CareCoach	Beyond training: coaches organising and reorganising themselves and others to deliver CareCoach sessions
Initiation	The work done by coaches to drive the implementation of CareCoach	Time requirements to build and sustain the implementation of CareCoach
Legitimation	The relational work of ensuring that coaches believe it is right for them to be involved in delivering CareCoach and that they can make a valid contribution	Am I right for the role? Coaches’ legitimation of their involvement in delivering CareCoach
Activation	Defining actions and procedures needed to sustain the delivery of CareCoach and to stay involved	The role of supervision sessions and other support in sustaining the delivery of CareCoach

**Table 4:** Definitions and themes found for sub-constructs of Collective Action.

NPT Sub-Constructs	Definition	Themes
Interactional Workability	The work done by coaches and carers or the work done by coaches using resources when operationalising CareCoach	Operationalising CareCoach: delivering sessions and maintaining carers engagement
Relational Integration	The knowledge work coaches did to build accountability and maintain confidence in implementing CareCoach	The role of peer support and experience in maintaining confidence in implementing CareCoach
Skillset Workability	Skills underpinning the allocation work of delivering CareCoach	The role of coaches’ skills and background in operationalising CareCoach

**Table 5:** Definitions and themes found for sub-constructs of Reflexive Monitoring.

NPT Sub-Constructs	Definition	Themes
Systematisation	The appraisal work done to determine how effective and useful CareCoach is for carers	CareCoach—empowering carers to manage their role and improve their wellbeing
Communal appraisal	The appraisal work by coaches and carers to evaluate CareCoach	Coaches’ and carers’ evaluation of CareCoach
Individual appraisal	The appraisal work done by coaches to evaluate how training and delivering CareCoach had affected them and the contexts in which they were set	CareCoach training and delivery enriching coaches’ usual practice
Reconfiguration	Coaches’ appraisal work that led to attempts to redefine CareCoach procedures or modify practices to refine intervention	Coaches’ appraisal of the blended delivery approach
		Coaches’ appraisal of training, resources and support structures

## Data Availability

The data presented in this study are available on request from the corresponding author due to confidentiality.

## Supplementary Materials

The following supporting information can be downloaded at: https://www.mdpi.com/article/10.3390/bs15081058/s1, File S1: Topic Guide for Interviews with Coaches.
