# Supplementary material for: Understanding the Implementation of CareCoach—A Blended eHealth Intervention for Carers of People Living with Dementia: A Qualitative Process Evaluation Using Normalisation Process Theory
Source: Behav Sci (Basel). 2025 Aug 5;15(8):1058. doi: 10.3390/bs15081058 (PMC12382862; doi:10.3390/bs15081058)
Supplement: Supplementary file 1 [file behavsci-15-01058-s001.zip › behavsci-3618557-supplementary.pdf]

CARECOACH

Topic Guide for Interviews with Coaches (and staff supporting or supervising coaches)

This Interview Schedule highlights the ***general topics*** for the interviewers to explore during the semi-structured interviews with carers. It is ***important to note*** that these interviews are ***semi-structured*** in design and hence the interviewer is in large part guided by the interviewee regarding both the specific areas covered and the emphasis given to particular topics. Topics may change and evolve due to the nature of the themes emerging from previous interviews.

Introductions

- Explain the purpose of the interview is to find out more about your experience of the CareCoach resource and coaching sessions. It will last up to an hour, and you are welcome to say when you need a break.
- Confirm verbal consent for interview including video/audio recording

Start video/audio recording

Collecting basic history of coaching experience

- How long have you been supporting people with dementia?
- How long have you been a coach? How have you found being a coach?
- Can you tell me about the approach you take to coaching?
- What have you found most rewarding/ frustrating?

Research process

- Can you talk me through how you came to be part of this study and how you felt about this?
- What did you think of the training to deliver the intervention?
- How did you feel about a trial such as this – how have the people you have been coaching found being part of this trial?
- Are there aspects of the trial you think people are finding difficult – do you have any suggestions how to address this?

CareCoach

Overall impressions

- What do you think about the approach of combining coaching and a web-based resource? Do you think these aspects work well together?

- Did you find the resources easy to use – why do you think that is?
- How did you find the meetings with the person you were coaching? What went well?
- Did you find working with the coaching resources difficult in any way?
- Has taking part turned out as you expected in terms of time/ in terms of what you have done? (if not, what was different and how did you feel about it?)
- Has there been any aspect of taking part that you weren't expecting?'

Coherence

- Have you found the resources helped in your wider work role?
- In what ways has it been different? Or not?
- Do you find this approach has added anything to your knowledge that you did not get from other sources of information? Why is that?

Barriers to use of CareCoach web programme

- How much time did you spend getting familiar with using the CareCoach web programme?
- Is it easy to find time to use it?

Facilitators of use of CareCoach web programme

- Was there anything at the start, that helped you to get going with CareCoach? What tips would you give to anyone new to it?
- Is there anything else that might support your use of the web-based programme? How could this help you?

Future use/use by others

- 'Would it be practical for you to use carecoach with clients?'
- 'Do you think you could commit to using it with clients regularly?
- Have you spoken about it with any work colleagues? Do you think they would find it useful?

Any other feedback?

- For us, going forward, is there any advice you'd give that you think would improve the way CareCoach is introduced or presented?

Concluding the interview

- Reiterate the boundaries of the interview
- What happens next
- Confirm details for sending voucher for participating
- Thank you and goodbyes
